# Occurrence and Nature of Double Alleles in Variable-Number Tandem-Repeat Patterns of More than 8,000 Mycobacterium tuberculosis Complex Isolates in The Netherlands

**DOI:** 10.1128/JCM.00761-17

**Published:** 2018-01-24

**Authors:** Rana Jajou, Miranda Kamst, Rianne van Hunen, Carolina Catherina de Zwaan, Arnout Mulder, Philip Supply, Richard Anthony, Wim van der Hoek, Dick van Soolingen

**Affiliations:** a National Tuberculosis Reference Laboratory, Centre for Infectious Disease Control, National Institute for Public Health and the Environment (RIVM), Bilthoven, The Netherlands; b KNCV Tuberculosis Foundation, The Hague, The Netherlands; c University Lille, CNRS, INSERM, CHU Lille, Institut Pasteur de Lille, U1019, UMR 8204, CIIL, Centre d'Infection et d'Immunité de Lille, Lille, France; d Radboud University Medical Centre, Department of Medical Microbiology, Nijmegen, The Netherlands; Carter BloodCare & Baylor University Medical Center

**Keywords:** Mycobacterium tuberculosis, VNTR, double alleles, The Netherlands

## Abstract

Since 2004, variable-number tandem-repeat (VNTR) typing of Mycobacterium tuberculosis complex isolates has been applied on a structural basis in The Netherlands to study the epidemiology of tuberculosis (TB). Although this technique is faster and technically less demanding than the previously used restriction fragment length polymorphism (RFLP) typing, reproducibility remains a concern. In the period from 2004 to 2015, 8,532 isolates were subjected to VNTR typing in The Netherlands, with 186 (2.2%) of these exhibiting double alleles at one locus. Double alleles were most common in loci 4052 and 2163b. The variables significantly associated with double alleles were urban living (odds ratio [OR], 1.503; 95% confidence interval [CI], 1.084 to 2.084; *P* = 0.014) and pulmonary TB (OR, 1.703; 95% CI, 1.216 to 2.386; *P* = 0.002). Single-colony cultures of double-allele strains were produced and revealed single-allele profiles; a maximum of five single nucleotide polymorphisms (SNPs) was observed between the single- and double-allele isolates from the same patient when whole-genome sequencing (WGS) was applied. This indicates the presence of two bacterial populations with slightly different VNTR profiles in the parental population, related to genetic drift. This observation is confirmed by the fact that secondary cases from TB source cases with double-allele isolates sometimes display only one of the two alleles present in the source case. Double alleles occur at a frequency of 2.2% in VNTR patterns in The Netherlands. They are caused by biological variation rather than by technical aberrations and can be transmitted either as single- or double-allele variants.

## INTRODUCTION

The Netherlands is a country with a low slowly declining tuberculosis (TB) incidence, which was 5.2 per 100,000 inhabitants in 2016 (1). However, due to the influx of asylum seekers, there were increases in the notification rate of 6% in 2015 and 3% in 2016. The national tuberculosis reference laboratory in The Netherlands, based at the National Institute for Public Health and the Environment (RIVM), performs DNA fingerprinting of all Mycobacterium tuberculosis complex isolates cultured in The Netherlands to monitor the transmission of TB. From 1993 to 2008, IS*6110* restriction fragment length polymorphism (RFLP) typing was used (2). This method was time consuming and technically demanding. Therefore, in 2009, 24-locus-based variable-number tandem-repeat (VNTR) typing was introduced as the new standard DNA fingerprinting method for newly obtained M. tuberculosis complex isolates, and retrospective VNTR typing was applied to all isolates from 2004 to 2008 (3). VNTR genotypes consist of a 24-number combination, reflecting the number of tandem repeats identified at each locus after size analysis of PCR amplicons from 24 target regions within the genome of M. tuberculosis (3, 4). Isolates that share identical 24-locus VNTR patterns are considered to be clustered. Municipal health services (MHSs) investigate TB cases clustered on the basis of VNTR patterns using contact information from patients gathered during interviews to study and control the transmission of this communicable disease.

Since 2009, the European Centre for Disease Prevention and Control (ECDC) has performed a yearly international proficiency study on VNTR typing in Europe and outsourced this to the RIVM to test the international intra- and interlaboratory reproducibility of VNTR typing. The results revealed an average of 72% intralaboratory reproducibility and 60% interlaboratory reproducibility in the first round in 2009 (4), which increased to 78% and 88%, respectively, in the second round in 2010 (5). Apart from other challenges, especially the analysis of loci with double alleles, there is a high degree of variation in the interpretation of VNTR patterns. This phenomenon consists of the occasional detection of two amplicons with slightly different sizes for one or more particular locus/loci, corresponding to distinct numbers of repeats (6). Often, technical challenges in calling the number of repeats and/or the PCR are proposed as explanations for these confusing results in VNTR typing. Double alleles at a single locus are also often assumed to reflect ongoing microevolution in two subpopulations within one clonal isolate on the basis of recurrent observations of sporadic single-locus variations in VNTR genotypes among longitudinal isolates from infected individuals or from human-to-human transmission (3, 6, 7). On the same basis, double alleles seen in two or more loci are considered evidence for mixtures of two independent strains (3, 8–16). However, whether double alleles truly represent such biological phenomena or are the result of VNTR pattern interpretation errors is still a matter of debate, especially when a single locus is affected without a concordant observation of double alleles at loci from other (multiplex) PCRs.

This study was initiated to quantify the prevalence of double alleles in a large collection of VNTR patterns produced according to the international standard and to determine their associated characteristics. On the basis of the availability of information on epidemiological links between the cases in The Netherlands, the transmission of double allele-containing strains was investigated. To examine the nature of the phenomenon of double alleles, single colonies from selected strains with doubles alleles were analyzed to see whether individual subpopulations could be segregated. If single colonies could be identified with different single alleles, this would confirm the hypothesis that biological mixture as opposed to technical artifacts/errors was the correct explanation in these cases.

## MATERIALS AND METHODS

### Study population.

M. tuberculosis complex isolates from clinical material of TB patients cultured between 1 January 2004 and 31 December 31 2015 were included. Patients with Mycobacterium bovis bacillus Calmette-Guérin (BCG), nontuberculous mycobacterium isolates, and confirmed laboratory cross-contaminations, i.e., isolates with identical VNTR patterns that were received within a 1-week time interval from the same peripheral laboratory, were excluded.

### Testing double alleles in single colonies.

To analyze the possible segregation of double alleles in single colonies, two random double-allele isolates from 2016 were selected. Bacteria were cultured from the −70°C freezer collection in a mycobacterial growth indicator tube for two and a half weeks and were thereafter smeared on a Middlebrook 7H10 agar plate. From separate colonies, lysates were prepared and VNTR typing was performed on the loci affected by double alleles in the parental strain.

### DNA fingerprinting.

VNTR typing was performed according to the international standard described by Supply et al. (3), and in 2015, a switch was made to an evolved version according to de Beer et al. (17). Gene Marker software version 1.51 was used for allele calling of the VNTR patterns.

In addition, whole-genome sequencing (WGS) was performed on the two randomly selected double-allele isolates from 2016 to compare single-colony cultures harboring separated alleles with their respective parental strains.

### Data collection and analysis.

The Netherlands Tuberculosis Register (NTR) is an anonymized database that contains information on many patient characteristics, such as treatment outcomes and epidemiological links, and is collected through continuous surveillance of all identified TB patients in The Netherlands. From the NTR, information regarding ethnicity, risk groups, urban (Amsterdam, Rotterdam, The Hague, and Utrecht)/rural living, having pulmonary tuberculosis (PTB)/extra pulmonary TB (ETB), the country of birth, BCG vaccination, a prior diagnosis of TB, prior treatment of latent tuberculosis infection (LTBI), immunosuppression, and epidemiological links was collected. Specific data regarding who infected whom have been available in the NTR since 2014; therefore, MHSs were contacted by phone to collect these data.

Statistical analysis was performed using SPSS version 22.0. A chi-square test was used to analyze the significance between independent variables and having double alleles, and a Fisher's exact test was applied when cell sizes were below five. Variables with *P* values of <0.2 were subjected to a univariate analysis in which odds ratios (ORs) including 95% confidence intervals (CIs) were calculated using the binary logistic regression method. Variables that had *P* values of <0.2 in univariate analyses were included in the backward prediction model to identify potential predictors for double alleles. A *P* value of <0.05 was considered to be statistically significant. WGS was performed on an Illumina HiSeq 2500 sequencer. Breseq software version 0.28.1 was used for mapping the raw sequence data against the H37Rv reference genome (GenBank accession number AL123456.3) and for calling single nucleotide polymorphisms (SNPs) using a minimum allele frequency of 80% in regions with a minimum coverage of five reads. R statistics version 3.3.2 was used for WGS data analysis, excluding genetic regions annotated as PE/PPE, PGRS, pks, esx, repeat, polyketide, and transposase.

## RESULTS

### Prevalence of double alleles.

During the period from 1 January 1 2004 to 31 December 2015, a total of 8,532 isolates from 8,458 TB patients were included in the study. Of these 8,532 isolates, 186 (2.2%) isolates from 185 unique patients were recorded as having double alleles at a single locus in VNTR typing. NTR data were available for 8,210/8,458 patients (97.1%). Patients with available NTR data did not significantly differ from patients with missing NTR data with respect to the variables presented in Table 1 (data not shown).

**TABLE 1:** Comparison of characteristics of 8,458 TB cases with regular and double-allele M. tuberculosis complex isolates

Characteristic	Patients without double-allele isolates (*n* = 8,273)^*a*^	Patients with double-allele isolates (*n* = 185)^*a*^	*P* value
Age (yrs) (*n* [%])			0.006
<15	196 (2.4)	2 (1.1)	
15–24	1,461 (17.7)	22 (11.9)	
25–34	2,137 (25.8)	37 (20)	
35–44	1,425 (17.2)	34 (18.4)	
45–54	1,043 (12.6)	23 (12.4)	
55–64	709 (8.6)	26 (14.1)	
≥65	1,301 (15.7)	41 (22.2)	
Missing	1 (0.01)	0 (0)	
Age (yrs) (mean [range])	41.4 (0–101)	47.1 (11–94)	
Sex (*n* [%])			
Male	4,898 (59.2)	112 (60.5)	0.934
Missing	44 (0.5)	1 (0.5)	
Resistance (*n*/total no. [%])			
To at least one antibiotic	1,034/7,571 (13.7)	18/166 (10.9)	0.308
Isoniazid	601/7,520 (8)	8/165 (4.8)	0.139
Rifampin	160/7,523 (2.1)	4/164 (2.4)	0.579
Ethambutol	84/7,522 (1.1)	1/164 (0.6)	>0.999
Pyrazinamide	234/7,507 (3.1)	2/164 (1.2)	0.247
Streptomycin	504/7,524 (6.7)	11/164 (6.7)	0.996
Species or subspecies (*n* [%])			0.482
M. tuberculosis	7,124 (86.1)	156 (84.3)	
M. africanum	117 (1.4)	3 (1.6)	
M. bovis	136 (1.6)	1 (0.5)	
M. canettii	3 (0.04)	0 (0)	
M. caprae	2 (0.02)	0 (0)	
M. microti	1 (0.01)	0 (0)	
MTBC^*b*^	890 (10.8)	25 (13.5)	

a Multiple TB episodes from the same patient were excluded.

b MTBC, Mycobacterium tuberculosis complex; MTBC represents isolates belonging to the M. tuberculosis complex, but not assigned to a (sub)species in The Netherlands Tuberculosis Register.

The percentages of males were almost equal among the isolates with and those without double alleles (60.5% versus 59.2%, respectively; *P* = 0.934). Patients with double alleles were significantly more frequently observed in the higher age categories than patients without double alleles (*P* = 0.006) and were more likely to be native Dutch (28.1% versus 19.7%, respectively; *P* = 0.013), be addicted to drugs (5.1% versus 2.4%, respectively; *P* = 0.023), live in an urban setting (41% versus 34.2%, respectively; *P* = 0.060), and have PTB (64.6% versus 52.1%, respectively; *P* = 0.001). The resistance to first-line antibiotics, although not significantly associated, was higher among isolates without double alleles, except for rifampin (Tables 1 and 2).

**TABLE 2:** Characteristics of 8,210/8,458 TB patients with available data in The Netherlands Tuberculosis Register

Characteristic^*a*^	No. (%) of patients^*b*^		*P* value
	Without double-allele isolates (*n* = 8,032)	With double-allele isolates (*n* = 178)	
Ethnicity			0.013
Native Dutch	1,580 (19.7)	50 (28.1)	
Non-native Dutch	6,294 (78.4)	123 (69.1)	
Unknown	158 (2)	5 (2.8)	
Risk group			
Contact	518 (6.4)	10 (5.6)	0.655
Immigrant	896 (11.2)	22 (12.4)	0.614
Refugee	903 (11.2)	11 (6.2)	0.034
Illegal	310 (3.9)	5 (2.8)	0.470
Homeless	174 (2.2)	3 (1.7)	>0.999
Alcohol addict^*c*^	138 (1.7)	2 (1.1)	0.772
Drug addict^*d*^	192 (2.4)	9 (5.1)	0.023
Prisoner^*e*^	225 (2.8)	4 (2.2)	0.820
Health/welfare worker	53 (0.7)	2 (1.1)	0.453
Old patient/recoded	425 (5.3)	12 (6.7)	0.394
Seafarer	9 (0.1)	0 (0)	>0.999
Traveler from/in areas of endemicity (>3 mo)	221 (2.8)	3 (1.7)	0.388
Documented contact with infectious patient	411 (5.1)	7 (3.9)	0.477
Urban living	2,750 (34.2)	73 (41)	0.060
Diagnosis			0.001
PTB	4,184 (52.1)	115 (64.6)	
ETB	2,759 (34.4)	38 (21.3)	
PTB+ETB	1,089 (13.6)	25 (14)	
Country of birth			0.174
Afghanistan	136 (1.7)	1 (0.6)	
China	126 (1.6)	5 (2.8)	
Eritrea	211 (2.6)	1 (0.6)	
Ethiopia	119 (1.5)	3 (1.7)	
Philippines	108 (1.3)	3 (1.7)	
India	196 (2.4)	4 (2.2)	
Indonesia	381 (4.7)	12 (6.7)	
Morocco	734 (9.1)	19 (10.7)	
The Netherlands	2,157 (26.9)	60 (33.7)	
The Netherlands-Antilles	81 (1)	1 (0.6)	
Pakistan	127 (1.6)	2 (1.1)	
Poland	84 (1)	8 (4.5)	
Portugal	36 (0.4)	1 (0.6)	
Russia	27 (0.3)	2 (1.1)	
Sierra Leone	82 (1)	2 (1.1)	
Somalia	1,098 (13.7)	12 (6.7)	
Suriname	345 (4.3)	8 (4.5)	
Turkey	251 (3.1)	7 (3.9)	
Vietnam	89 (1.1)	2 (1.1)	
Other	1,644 (20.5)	25 (14)	
BCG vaccination			0.983
Yes	2,315 (28.8)	51 (28.7)	
No	1,803 (22.4)	41 (23)	
Unknown	3,914 (48.7)	86 (48.3)	
Prior diagnosis of TB			0.463
Yes	453 (5.6)	12 (6.7)	
No	6,842 (85.2)	154 (86.5)	
Unknown	737 (9.2)	12 (6.7)	
Prior treatment for LTBI^*f*^			0.210
Yes	229 (3.2)	6 (3.8)	
No	6,346 (87.7)	143 (91.1)	
Unknown	657 (9.1)	8 (5.1)	
Immunocompromised			
HIV positive	337 (4.2)	10 (5.6)	0.336
Diabetes	346 (4.3)	7 (3.9)	0.807
Malignancy	194 (2.4)	5 (2.8)	0.736
Renal failure/dialysis	102 (1.3)	3 (1.7)	0.498
Organ transplantation	27 (0.3)	1 (0.6)	0.459

a PTB, pulmonary TB; ETB, extrapulmonary TB; BCG, bacillus Calmette-Guérin; LTBI, latent TB infection; HIV, human immunodeficiency infection.

b Multiple TB episodes from the same patient were excluded.

c Defined in The Netherlands Tuberculosis Register as being a problematic alcohol user when TB was diagnosed, i.e., leading to psychical and/or psychiatric or social problems, which prevents problems from being solved in an adequate manner.

d Defined in The Netherlands Tuberculosis Register as using hard drugs (including methadone and cocaine) on a regular basis, which results in social derailment.

e Defined in The Netherlands Tuberculosis Register as a person who was in a prison at the time of TB diagnosis.

f Percentages are based on totals of 7,232 patients without double-allele isolates or 157 patients with double-allele isolates.

### Predictors of double alleles.

Seventy-two patients had multiple TB episodes, which were excluded from the logistic regression analysis. From the remaining 8,458 isolates, 1,155 (13.7%) had incomplete NTR data for all variables analyzed in the multivariate analysis combined, leaving 7,303 isolates to be included in the multivariate model. The factors age (in categories), being native Dutch, being addicted to drugs, having PTB, living in an urban setting, showing isoniazid resistance, and being a refugee had *P* values of <0.2 in univariate analyses and were added to a backward prediction model to analyze predictors for double alleles at a single locus. The variable sex was also added in the prediction model, although it had a *P* value greater than 0.2. The final multivariate model consisted of the factors urban living and having PTB (Table 3).

**TABLE 3:** Univariate and multivariate analyses for potential predictors of double alleles

Variable	Univariate analysis^*a*^		Multivariate model^*b*^	
	OR (95% CI)^*c*^	*P* value	OR (95% CI)	*P* value
PTB^*d*^	1.679 (1.231–2.290)	0.001	1.703 (1.216–2.386)	0.002
Urban living	1.335 (0.987–1.807)	0.061	1.503 (1.084–2.084)	0.014
Age (yrs)				
<15	Reference			
15–24	1.476 (0.344–6.324)	0.600		
25–34	1.697 (0.406–7.093)	0.469		
35–44	2.338 (0.557–9.809)	0.246		
45–54	2.161 (0.505–9.240)	0.299		
55–64	3.594 (0.846–15.273)	0.083		
≥65	3.088 (0.741–12.870)	0.121		
Male sex	1.000 (0.998–1.002)	0.989		
Native Dutch	1.619 (1.160–2.260)	0.005		
Drug addict	2.175 (1.096–4.316)	0.026		
Isoniazid resistance	0.587 (0.287–1.199)	0.144		
Refugee	0.520 (0.281–0.961)	0.037		

a In total, 8,210 TB patients with available data in The Netherlands Tuberculosis Register were included in the univariate analysis.

b In total, 7,303 patients had complete data in The Netherlands Tuberculosis Register for the variables age, PTB, urban living, native Dutch, drug addict, isoniazid resistance, and refugee and were included in the multivariate model.

c OR, odds ratio; CI, confidence interval.

d PTB, pulmonary tuberculosis.

### Frequencies of double alleles and allelic variability by locus.

**FIG 1:**
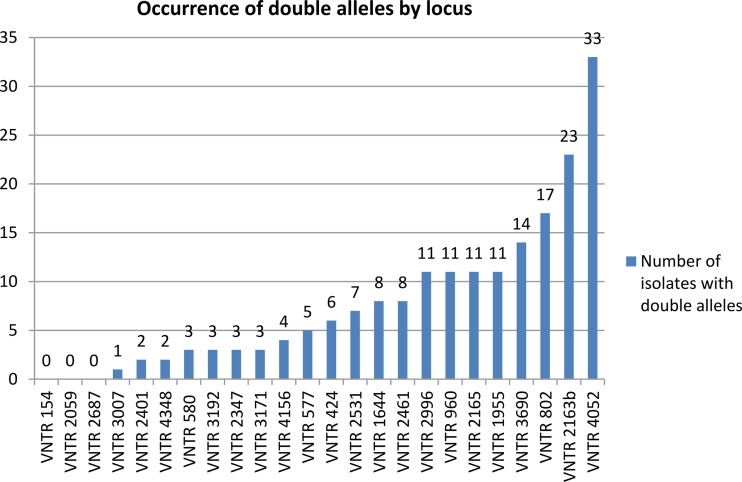
Occurrence of double alleles by locus (*n* = 186).

**FIG 2:**
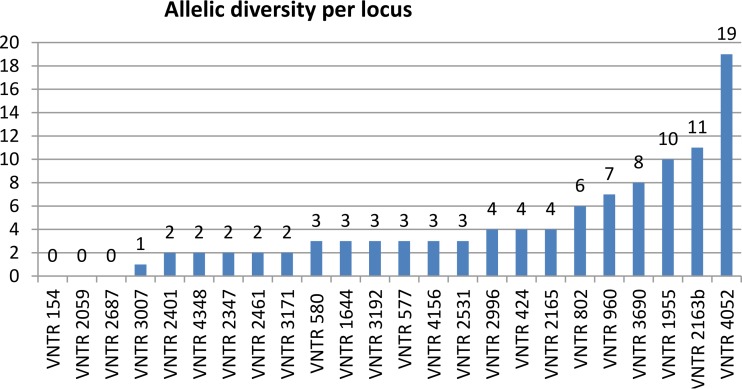
Allelic diversity per locus within double-allele isolates (*n* = 186).

The frequency of double alleles by locus was analyzed. No double alleles were observed at locus 154, 2059, or 2687. The loci most affected by double alleles were 4052 and 2163b (Fig. 1). The largest variations in the numbers of repeats between alleles within any double-allele case were observed for loci 4052, 2163b, and 1955 (Fig. 2).

### Transmission of double-allele strains in a subset of the study population.

Ninety-one of 186 double-allele isolates (48.9%) were part of 86 clusters, comprising between 2 and 134 isolates. Of these 86 clusters, 16 clusters included two or more isolates with double alleles, but no cluster was composed solely of double-allele isolates.

There were three clusters with epidemiological links confirmed by MHSs and at least one double-allele case. In the first cluster, a double-allele (at locus 1644) source case infected two patients; one patient had a VNTR pattern with double alleles but at another locus (4052), and a second patient had only a single allele at locus 1644, which matched one of the two alleles in the source case. The second cluster consisted of a double-allele (at locus 2461) source case that transmitted the strain to four other patients, all with the same double alleles. The third cluster included a single allele index patient that infected a secondary case with the same VNTR pattern, except that it had a second allele at locus 3690 in addition to the allele shared with the source case. This secondary double-allele case infected a new patient, whose isolate displayed the same full single-allele profile as the source patient.

### Evolution from single to double alleles or vice versa within a patient.

Twenty-five patients had two M. tuberculosis isolates, with one patient showing double alleles in both isolates (one isolate had one and two repeats at locus 4052 and the other isolate had one and six repeats at locus 2163b) and one patient having completely different VNTR patterns in the first and second isolates, suggesting reinfection or sampling/laboratory errors. Isolates from the remaining 23 patients showed single alleles in one of the isolates and double alleles at one locus in the other isolate. For 12/23 patients (patients 1 to 12), the first isolate revealed a single allele and the second isolate showed double alleles at one locus. For the 11 remaining patients, the first isolate revealed double alleles and the second isolate showed a single allele at one locus (see Table S1 in the supplemental material).

In all cases, the single allele matched one of the two alleles seen in the paired isolate. In 15/23 (65.2%) cases, the difference in the number of tandem repeats between the single and double alleles consisted of a single repeat unit change. In 9/12 (75%) patients with double alleles in the second isolate, the number of tandem repeats decreased in the latter isolate. Likewise, in 7/11 (63.6%) of the remaining patients, the number of tandem repeats in the variant allele isolate was lower than in the single allele isolate (Table S1).

### Single-colony culture analysis of two double-allele strains.

From two double-allele M. tuberculosis strains, isolated in 2016, single colonies were generated. From one strain, two colonies were analyzed. While two amplicons representing two and four repeats at locus 424 were obtained from the corresponding parental strain, a single amplicon of two repeats was obtained from one colony and a single amplicon of four repeats was generated from the other one. When WGS was applied, a genetic distance of a maximum of three SNPs was observed between isolates of single colonies and the parental strain.

From the other parental strain with double alleles consisting of nine and ten repeats at locus 960, eight single colonies were subjected to VNTR analysis. Seven of eight revealed only a single amplified fragment of ten repeats, and one colony had a fragment comprising nine repeats at locus 960. WGS revealed a genetic distance of a maximum of five SNPs between the single colony isolates and the parental strain.

## DISCUSSION

We report the first large population-based study to investigate the occurrence of double alleles in VNTR patterns in detail. In our database consisting of more than 8,000 M. tuberculosis complex isolates from 2004 to 2015, we found that 2.2% of isolates presented double alleles at a single locus of their VNTR patterns. Different lines of evidence, including results from our analyses of single colonies with associated WGS data and of confirmed clusters of recent transmission, indicate that such double alleles reflect real genetic drift in most cases rather than technical or interpretation errors. Moreover, we found that the occurrence of such drift in VNTR patterns was consistently associated with particular patient factors, putatively reflecting longer periods of bacterial evolution/disease incubation within the patients. Compared to those of several previous studies, the large sample size of this study is important, as it increases the reliability and validity of the prevalence study as well the detection of potential associations. Furthermore, extensive epidemiological data, which were collected in a consequent manner in the NTR for all TB patients in The Netherlands, were available for the included patients.

It is unlikely that the double alleles identified in our study are due to technical errors. In our laboratory, when double alleles were observed at one locus, the PCR amplification of that specific locus was always repeated separately for confirmation. We follow well-established rules for correctly distinguishing these alleles from so-called stutter peaks representing typical PCR artifacts seen with VNTR markers (3, 15, 18). Moreover, positive biological evidence was obtained. Consistent with previous observations (10), we found that double alleles in selected parental strains could be segregated into single alleles in single colonies, which represents direct evidence for the coexistence of clonal variants/subpopulations within the parental strains. In addition, repeated transmission of such two-clonal variants/double alleles from one source case to multiple secondary cases was consistently detected in at least one cluster in our study population. However, in other instances, only one variant/one of the two alleles was apparently transmitted. As hypothesized, for the detection of independent strains among sputum samples from mixed-infection cases (13), such differential transmission plausibly reflects differential opening of separate lesions containing distinct clonal subpopulations in the lungs during the collection of the clinical specimens from the patients and during transmission between patients.

Previous studies have shown that isolates with identical VNTR patterns can be genetically distinct when WGS is applied (19–27). When WGS was applied to two double-allele samples from this study, fewer than 5 SNPs were observed between single-allele and double-allele isolates from the same patient, which makes it more likely that the single- and double-allele isolates derive from the same strain. However, although extremely coincidental, there remains a possibility that a proportion of double-allele isolates identified represent mixed infections from two highly similar strains.

Differences were seen in the prevalence of double alleles at one locus among studies from different settings. While the prevalence of such alleles in this study and another population-based study with more than 800 isolates from the Brussels region (28) ranged from approximately 1% to 2%, Huyen et al. (11) found that the occurrence of double alleles at one locus was almost 5% (60/1,248) of the isolates when using 15-locus VNTR typing in a population-based study in rural Vietnam. It is conceivable that differences in case finding and treatment exist among these distinct patient populations, similarly impacting the mean sizes and the diversification time of bacterial populations within individual hosts.

Loci for which double alleles were most (4052 and 2163b) or least (154, 2059, and 2687) frequently found were also those with the highest or the lowest allelic diversities in this strain population. Results from analyses of other strain collections are fully in line with our findings. For instance, loci 4052 and 2163b showed the highest diversities among the 24 loci when evaluated for standardization both on a global collection of 494 strains of diverse genetic lineages (3) and on a global panel composed of 535 Beijing strains only (6). These loci were likewise most affected by double alleles in the same global panel of Beijing strains (6) as well as in a population-based collection of 807 isolates in Belgium (28). This positive correlation between frequencies of double alleles linked to microevolution within individual isolates and allelic diversities of loci in strain populations is expected, as both parameters are predicted to depend upon differences in mutation rates of the different markers. Interestingly, while most of the other markers are intergenic, loci 4052 and 2163b correspond to repeats within sequences encoding a hypothetical arginine and proline rich protein (*Rv3611*) and a PPE protein (*Rv1917c*, alias *PPE34*), respectively (29, 30). Whether the apparently higher rates of (micro)evolution of these loci reflect positive selection linked to antigenic variation or to other virulence-related functions of such proteins remains to be investigated.

Somewhat unexpectedly, a detailed analysis of available microbiological and patient data over this near-comprehensive population-based data set revealed associations between double-allele isolates and particular patient factors. Cases with double-allele isolates were significantly more likely to be ≥55 years, drugs addicts, native Dutch, living in an urban setting, and to have PTB than patients without double-allele isolates. At least some of these associations are explainable. As disease in older Dutch residents tends to be the result of endogenous reactivation (31), an association with older age might reflect increased drift linked to longer periods of subclinical incubation of bacterial populations. In addition, reactivation itself may be associated with changes in DNA expression and genetic rearrangements. Of note, this type of genetic rearrangement is in agreement with the higher frequency in occurrence of low-intensity bands in IS*6110* RFLP patterns in isolates from the elderly found previously in The Netherlands (32). A similar explanation probably holds for the association with drug addicts, as drug users tend to wait longer to present for diagnosis and treatment after TB symptom onset (33), resulting in more advanced stages of disease and presumably larger bacterial populations with more potential for diversification. This was confirmed by the detection of twice as many smear positives (*P* < 0.001) among drug addicts compared to that among non-drug users in our study (data not shown).

In conclusion, our findings, obtained by using the largest population-based data set ever investigated for this phenomenon, supports the conclusion that the detection, under controlled technical conditions, of single-locus double alleles in an M. tuberculosis complex isolate most often reflects ongoing microevolution within a clonal infection. Although reporting the relatively rare detection of single-locus double alleles in a strain complicates interpretation by the MHSs, we do report both allele variants, as we are convinced that this phenomenon has a biological basis and such isolates may be linked to cases with either or both VNTR profiles observed. This approach is supported by our observations of the occasional documented transmission of single-locus variants from double-allele source cases.

## Supplementary Material

Supplemental material
