# Supplementary material for: Occurrence and Nature of Double Alleles in Variable-Number Tandem-Repeat Patterns of More than 8,000 Mycobacterium tuberculosis Complex Isolates in The Netherlands
Source: J Clin Microbiol. 2018 Jan 24;56(2):e00761-17. doi: 10.1128/JCM.00761-17 (PMC5786718; doi:10.1128/JCM.00761-17)
Supplement: Supplemental material [file supp_56_2_e00761-17__index.html]

Supplemental material 

# Occurrence and Nature of Double Alleles in Variable-Number Tandem-Repeat Patterns of More than 8,000 Mycobacterium tuberculosis Complex Isolates in The Netherlands

## Supplemental material

- Supplemental file 1 -

  Table S1 (Evolution from single to double alleles or vice versa within a patient)

  PDF, 294K
