# Supplementary material for: Occurrence and Nature of Double Alleles in Variable-Number Tandem-Repeat Patterns of More than 8,000 Mycobacterium tuberculosis Complex Isolates in The Netherlands
Source: J Clin Microbiol. 2018 Jan 24;56(2):e00761-17. doi: 10.1128/JCM.00761-17 (PMC5786718; doi:10.1128/JCM.00761-17)
Supplement: Supplemental material [file JCM.00761-17_zjm999095805s1.pdf]

Table S1 Evolution from single to double alleles or vice-versa within a patient

Patient	Sample	Time interval between isolates	VNTR 580	VNTR 2996	VNTR 802	VNTR 960	VNTR 1644	VNTR 3192	VNTR 424	VNTR 577	VNTR 2165	VNTR 2401	VNTR 3690	VNTR 4156	VNTR 2163b	VNTR 1955	VNTR 4052	VNTR 154	VNTR 2531	VNTR 4348	VNTR 2059	VNTR 2687	VNTR 3007	VNTR 2347	VNTR 2461	VNTR 3171
**1**	**04-0393**	8 days	5	2	3	4	3	4	2	2	9	2	5	1	4	11	4	2	2	1	2	2	3	3	4	3
	**04-0613**		5	2	3	4	3	4	2	2	9	2	5/2	1	4	11	4	2	2	1	2	2	3	3	4	3
**2**	**06-1469**	32 days	2	7	3	6	4	4	4	2	3	2	3	4	2	4	8	2	5	3	2	1	3	4	2	3
	**06-1745**		2	7	3	6	4	4	4	2	3/2	2	3	4	2	4	8	2	5	3	2	1	3	4	2	3
**3**	**08-1234**	67 days	2	5	3	5	1	3	2	3	3	4	1	3	6	3	6	2	5	2	2	1	3	4	2	3
	**08-1466**		2	5	3	5	1/3	3	2	3	3	4	1	3	6	3	6	2	5	2	2	1	3	4	2	3
**4**	**09-0041**	18 days	3	4	4	3	4	3	3	4	4	2	3	2	4	1	7	2	5	2	2	1	3	4	2	3
	**09-0276**		3	4	4	3	4	3	3	4	4	2	3	2	4	1	7	2	5	2	2	1	3	4	2	3/1
**5**	**09-1570**	139 days	5	2	2	4	2	6	2	4	7	2	2	1	5	4	6	2	6	2	2	2	3	3	6	3
	**10-0356**		5	2	2	4	2	6/5	2	4	7	2	2	1	5	4	6	2	6	2	2	2	3	3	6	3

**6**	**09-1759**	5 days	2	4	6	3	3	3	2	3	3	4	3	1	3	4	6	2	5	3	2	1	3	4	2	2
	**09-1644**		2	4	6/5	3	3	3	2	3	3	4	3	1	3	4	6	2	5	3	2	1	3	4	2	2
**7**	**09-1737**	1 day	2	1	2	4	1	3	1	4	3	4	3	3	1	3	7	2	5	2	2	1	3	4	2	3
	**09-1747**		2	1	2	4	1	3	1	4	3	4	3	3	1	3	7/6	2	5	2	2	1	3	4	2	3
**8**	**10-0535**	2 days	2	5	1	4	2	3	4	4	2	1	2	2	3	3	6	2	6	2	2	1	3	5	1	5
	**10-0490**		2	5/4	1	4	2	3	4	4	2	1	2	2	3	3	6	2	6	2	2	1	3	5	1	5
**9**	**10-0707**	33 days	2	4	5	3	1	3	5	5	3	2	5	2	3	2	5	2	5	2	2	1	3	4	2	3
	**10-0945**		2	4	5/6	3	1	3	5	5	3	2	5	2	3	2	5	2	5	2	2	1	3	4	2	3
**10**	**11-1013**	72 days	2	5	3	6	3	3	2	3	3	4	3	3	6	3	7	2	3	2	2	1	3	2	2	3
	**11-1222**		2	5	3	6	3	3	2	3	3	4	3	3	6/5	3	7	2	3	2	2	1	3	2	2	3
**11**	**12-1563**	834 days	2	5	4	3	1	3	2	4	3	2	5	2	4	2	5	2	5	2	2	1	3	2	2	3
	**15-0129**		2	5	4	3	1	3	2	4	3	2	5/7	2	4	2	5	2	5	2	2	1	3	2	2	3
**12**	**15-1171**	8 days	2	7	3	4	3	5	4	2	4	2	4	4	2	4	7	2	5	3	2	1	3	4	2	3
	**15-1169**		2	7	3	4	3	5	4	2	4	2	4/3	4	2	4	7	2	5	3	2	1	3	4	2	3
**13**	**04-0431**	7 days	2	4	1/3	2	3	3	3	4	3	2	4	2	2	3	7	2	5	2	2	1	3	4	1	3

	**04-0447**		2	4	3	2	3	3	3	4	3	2	4	2	2	3	7	2	5	2	2	1	3	4	1	3
**14**	**08-1192**	31 days	2	4	2	4/5	3	3	3	4	2	1	1	3	2	3	7	1	6	2	2	1	3	4	2	3
	**08-1369**		2	4	2	4	3	3	3	4	2	1	1	3	2	3	7	1	6	2	2	1	3	4	2	3
**15**	**08-1245**	61 days	2	4	3	4	3	3	2	4	2	1	4/5	2	2	3	7	2	6	2	2	1	3	4	2	3
	**08-1574**		2	4	3	4	3	3	2	4	2	1	4	2	2	3	7	2	6	2	2	1	3	4	2	3
**16**	**10-1377**	84 days	2	1	3	7	2	2	3	4/5	4	4	1	3	2	3	7	2	5	2	2	1	3	4	2	3
	**10-2036**		2	1	3	7	2	2	3	5	4	4	1	3	2	3	7	2	5	2	2	1	3	4	2	3
**17**	**11-0205**	3 days	2	5	2	3	4	3	2	3	2	2	3	2	4	2	2/3	2	5	2	2	1	3	4	2	2
	**11-0329**		2	5	2	3	4	3	2	3	2	2	3	2	4	2	3	2	5	2	2	1	3	4	2	2
**18**	**15-0854**	45 days	2	5	1/3	5	3	3	2	3	3	4	3	3	3	3	5	2	5	2	2	1	3	4	2	3
	**15-1009**		2	5	1	5	3	3	2	3	3	4	3	3	3	3	5	2	5	2	2	1	3	4	2	3
**19**	**16-0146**	0 days	2	1	4	7	4	4	4/2	2	4	2	2	4	2	3	3	2	5	3	2	1	3	4	2	3
	**16-0145**		2	1	4	7	4	4	4	2	4	2	2	4	2	3	3	2	5	3	2	1	3	4	2	3
**20**	**06-0503**	0 days	5	2	1	4	3	5	2	3	9	2	5	1	6	3/6	6	2	6	3	1	2	3	3	5	3
	**06-0736**		5	2	1	4	3	5	2	3	9	2	5	1	6	6	6	2	6	3	1	2	3	3	5	3

**21**	**06-1679**	0 days	2	7	2	4	3	4	4	4/5	2	1	5	2	3	2	4	2	4	2	2	1	3	4	2	3
	**06-1704**		2	7	2	4	3	4	4	4	2	1	5	2	3	2	4	2	4	2	2	1	3	4	2	3
**22**	**09-0561**	0 days	2	7	3	3	3	5	4	2	4	2	3	4	2	4/5	7	2	5	3	2	1	3	4	2	3
	**09-0388**		2	7	3	3	3	5	4	2	4	2	3	4	2	5	7	2	5	3	2	1	3	4	2	3
**23**	**09-0857**	0 days	2	5	3	4	3	3	4	3	3	4	4	3	2	3	8/7	2	5	2	2	1	2	4	2	3
	**09-0849**		2	5	3	4	3	3	4	3	3	4	4	3	2	3	8	2	5	2	2	1	2	4	2	3
